# Notes of an European Tour

**Published:** 1847-04

**Authors:** F. H. Hamilton


### BUFFALO MEDICAL JOURNAL

AND

### MONTHLY REVIEW.

**VOL. 2.** APRIL, 1847. **NO. 11**

### ORIGINAL COMMUNICATIONS.

ART. I.—*Notes of an European Tour.* By F. H. HAMILTON, M. D.,

At the depot opposite Aix-la-Chapelle my baggage was overhauled, weighed and marked, this being the frontier town of Prussia.—These small yet important matters arranged, I walked hastily towards the town, situated a short distance north of the railroad. Railroads are fatal enemies to ivy and romance. and one could almost wish they might never be allowed to disturb with their modern, mercenary tumult such venerable spots as Aix-la-Chapelle.—These springs, so famed for their sanative virtues, were well known to the Romans who conquered Gaul; and as they bubbled warm and sulphurous from the crevices of the rocks and from the heated sands, the worn soldier must have been pleasantly reminded of the volcanic soil and sunny air of his own far-off Latium, and have been tempted to lay aside his heavy armor to enjoy again, after so long a time, the luxuries of a thermal spring, not inferior to those of Baiae, of Pozzuoli, or of Solfatara. Of the correctness of our suspicion we have sufficient evidence in the numerous fragments of Roman baths &c., which have from time to time been here brought to light.

In this rugged, sulphurous place, Charlemagne was born, and here, some say, he died in 814, and was buried in the old *Dom*

*Kircke*, under a marble slab, upon which I read in large letters "Carolo Magno" and no more. It is something to be so vast that the name "great" shall never cease to distinguish you from all the world. "Carolo Magno!" what a sublime epitaph! I have little reverence however for Kings or Emperors, or any other of the race of tyrants, and felt no peculiar awe or alarm as I trod over the sepulchre of this great man. Possibly you may think I had not much reason to fear his anger, since not even his ashes are now here; with his pilgrims pouch and faithful sword Joyeuse, they were carried to Vienna by Otho.

But this old buildiug has not lost all its treasures. Listen while I read the catalogue.—Dried manna from the wilderness! A piece of Aaron's rod! A rib of St. Stephen, and some earth stained with his blood! A tooth of St. Catherine! Good old Simeon's right arm! The linen girdle worn by Christ when crucified, stained with blood! The chord which bound the rod which smote him! A piece of the cross and a nail from the same! The sponge which held the gall and vinegar, which same may be seen in the church of St. Croce in Gerusalemme at Rome! A locket of the Holy Virgin's hair! The white gown worn by her at the nativity! The swaddling clothes (a coarse sacking)! Precious relics, which together with the warm springs, render Aix-la-chapele the very fountain of health and life. The streets are lined with the halt and the lame, and blotched and scaly lepers, and as I pressed up through the crowd to the large fountain of Eliza, I thought that even the skin wards of St. Louis at Paris could scaiccly afford a better opportunity for the study of cutaneous diseases. This spring at its source has a temperature of 146° Farenheit, but at the fountain I should not think it more than 100°. It tastes strongly of sulphur and of certain salts, and is not unpleasant.

*Cologne*, or as its Roman founder named it, Colonia Agrippina, after Agrippina the mother of Nero, who was born here, is, as every body knows and every body writes, not the cleanest nor most sweetly scented city in the world, *malgre*, its half-a-dozen "original Jean Marie Farina's," its twenty-four manufactories and its ten thousand bottles of Eau de Cologne.

Cologne is about as large as Brooklyn, and stands on the left bank of the Rhine; the river being crossed at this point by a bridge of boats. The unfinished cathedral is the great marvel of the place. I never describe cathedrals. It was commenced in 1248, and it will be time enough for me to describe it when it is completed. But it contains one single shrine, the contents of which you would go far to see, the skulls of the three magi, Gaspar, Melchior and Balthazer, who came with presents from the east to visit the infant Saviour! In front of which shrine under a slab, lies the heart of Maria de Medicis, who ended her days in an old house in the street called Sternen Gasse, the same in which in 1577 Rubens was born.

I was just leaving this old *unfinished ruin*, when I met my *compagnon de voyage* Mr. Ed. Burchard, of Rostock, Mecklenburgh, whom I had left some months before at Rome, and whom I then never expected to see again, although we had promised to correspond with each other. He had gone from Rome to Venice and Vienna, and I had turned towards Marseilles and Paris and now from these remote and opposite points we met at the threshold of the old cathedral of Cologne. Mr. Burchard was the only man with whom I had travelled any considerable distance, and the only one with whom I had formed any special attachment during my absence, and we could but regard this as a most remarkable meeting. After a hearty shake of the hands and a german embrace, we walked home to my lodgings to recount onr varions adventures, our haps and mishaps since we parted on the steps of Hotel D'Allemagne at Rome. We spent a pleasant evening and separated, mutually agreeing to spend the next day together in our reconnoitre of Cologne.

Our resolution was piously kept, although we found our tastes for sight-seeing not wholly alike. B. was burning to see one of the master works of Rubens, his famous altar piece in the church of St. Peter, while I was caring little for Rubens or his pictures, but was urging him to accompany me to the very old and very dirty hospital of St. Cecilia. After all, a man whose business is information rather than pleasure, does best to travel alone.

Of course I want to see Rubens' picture, presented to the church in which he was baptized, and one of the last which he ever painted, and as he thought, his best. It is the crucifixion of St. Peter, with his head downwards. Burchard, to whose judgment upon pictures I always submit, says it is a good picture, but not his best.

I have paid B. well for detaining me so long at this old church, by taking him to the hospice, and I would not let him go for he was to serve as my interpeter. "It does not smell very fragrant here" said B. as we entered the large court. "No" I said "but about as fragrant as any where else in Cologne." The court is a sort of common sewer for the hospice.

We were led into one of the upper lofts amid odors of garlic and sour-krout, which steamed forth from the *salle a manger*, and introduced by an interne to M. Balderman, surgeon in chief of the hospital, a very kind, intelligent gentlemanly German. He complained to me of the bad arrangement of his wards, their imperfect ventilation, and the insufficiency of servants and means. The number of inmates is 350, and of patients, 134. I examined several surgical cases, but saw nothing of interest. The circular amputation, M. Balderman tells me, is made generally in Germany. He cures opacity of the cornea by blowing calomel into the eye with a quill. I enquired how Homœopathy flourished in Cologne. "I have not seen" said he "a Homœopathist in Cologne for several years past, but many years ago they were numerous; Hydropathy has now taken its place here, and throughout Germany, and this is again beginning to be supplanted by the "Grape cure." Crowds now flock to the Rhine during the vintage to cram themselves with grapes, and it is said to be a remedy for every disease.

As Mr. B. was now *en route* for Holland and I for Frankfort on the Main, we were compelled once more to part, but with a promise that we should meet each other just two weeks from the day we parted, at the Consul's house in Amsterdam.

From Cologne to Bonn I rode by the newly constructed railroad, and from thence to Mayence, through all the most picturesque part of the Rhine I travelled by steamer, this being the only way in which one can obtain a proper view of all its varied beauties.

From Bonn to Mayence nature has wrought the shores into the most rude and gothic carvings, into crags and dark chasms and sharp jutting promontories which almost cut the narrow river in twain; and art has appropriately embellished the scene by crowning each uplifted rock with some tower or fortress or feudal castle; time has also drawn over both tower and rock her dark grey colours, and blended them together, until you can scarce distinguish the one from the other: to all this, history and German romance has added a thousand associations,* until every island, valley, mountain and every crumbling ruin has its traditionary legend of fairies, nymphs, goblins, love, warrior knights and baronial feuds. I left Bonn in what might be called a third-rate North River boat, but one of the best boats on the Rhine, (the Cologne company), and during the twenty hours we were in reaching Mayence I do not think I left the deck five minutes, so wrapt have I been in its scenes of unrivalled beauty and grandeur.

On the right we passed first, the hoary basaltic mountain of Drachenfels.

"The castled crag of Drachenfels,
Towers o'er the wide and winding Rhine,
*    *    *    *    *    *    *
Here strewed a scene which I should see
With double joy, were *thou* with me."

On one of the neighboring hills, are seen the ruins of the castle of Lowenberg, where Melancthon spent considerable time. But alas! I have a thousand "castled crags" before me, which call up the "glories of old days;" my book is filled with notes and memorandums, and points of exclamation and admiration, and rude pencil sketches which, as I am reminded, the character and purpose of these communications forbids me to transcribe. You must before you die, see the Rhine yourself.

From Mayence, where was born Gutemberg, the inventor of movable types, I rode by a very excellent railroad to Frankfort on the Main, a place of about 60,000 inhabitants, of whom 5000 are Jews. It is called a *free town*—take care that you do not interpert the appellation literally, and utter aloud your pent up thoughts. If you *think* even of freedom in this place, let it be as privately as possible.

Goethe was born in Frankfort, in an old house still standing, and which may be recognized by the lyres over the door. In the Jew quarter, still lives also the mother of the Rothschilds, in a very antique looking little building, while her son the Baron, lives in a palace just without the walls. This is, however, her own choice.

A litttle back of the city on a rise of ground, is the city cemetery or "court of peace" as the Germans call it, where the celebrated physician, surgeon and naturalist Soemmering was buried but a few years since,

A custom prevails at Frankfort, and some other German cities, worthy of notice. Soon after death, the bodies of both rich and poor are conveyed to the cemetry and for a time deposited in a temporary sepulchre or a kind of chapel, hung with the emblems of mourning and lighted by a taper. And to prevent all possibility of burying a body while in a state of only suspended animation, the fingers of the corpse are attached to a string, which has suspended from its other extremity in an adjoining apartment a bell, so that the smallest motion of the finger will notify the guard, who is in constant attendance, of the return of life.

I have never felt much confidence in the marvellous cases which are occasionally reported, seldom upon the most reliable authority, of bodies buried alive, and of others who were scarcely saved from such a horrible fate by a timely recuscitation, yet I cannot deny its possibility, and the custom of which I have just spoken, by keeping the body disentombed until putrefaction has actually commenced, may be well worthy the imitation of all civilized and humane nations.

Heister, a celebrated army surgeon, was born here in 1683. He was author of a work entitled "Institutions of Surgery" and also of another entitled "Medical, Surgical and Anatomical cases and observations." He was finally appointed Physician, Aulic Counselor, and professor of medicine under the Duke of Brunswick at Helmstadt.

From Frankfort by railroad in two hours, we were at Weisbaden, in twenty minutes I was installed in the "Four Seasons" (I found only summer)—in half an hour I had taken a greasy-looking hot bath, of the temperature of 140° Farenheit, heat by a furnace lighted many centuries ago by that old stoker Pluto. In an hour I had breakfasted on coffee and toast to the tune of 30 *kratsen*, in an hour more I had contracted with a *lohnbedienter*, (or valet-de-place) and in two hours I had drank hot water enough from a dozen steaming fountains to make my stomach feel completely "overdone," and for the next half hour I amused myself and my valet by ineffectual attempts to dispose of the excess. For an hour more I wandered up and down looking into all curious and outlandish places, into cafes, and gardens, and billiard rooms and gambling houses and gazing at the multitude of animals of the sapajoo species, described by lord Monboddo, who were running loose every where with long beards, and little caps, and queer shaped overcoats, and pants of vasty dimensions, all of whom were espeeially distinguished by the long German pipe suspended from between their teeth—and I wondered most what rendered such eternal fumigation necessary. In an hour more I was at Mayence waiting for the steamer to float me down to Rotterdam.

